# External Validation of the Simplified Acute Physiology Score-3 in a Cohort of 5,033 Patients in Mexico

**DOI:** 10.7759/cureus.101652

**Published:** 2026-01-15

**Authors:** Santa López-Marquez, Pablo Álvarez-Maldonado, Ulises W Cerón-Díaz

**Affiliations:** 1 Intensive Care Unit, "Alberto Villazón S" Hospital Español, Mexico City, MEX

**Keywords:** critical care, intensive care, prediction model, saps-3, validation study

## Abstract

Background: Since its introduction, the Simplified Acute Physiology Score-3 (SAPS-3) has remained one of the most widely adopted scoring systems for mortality prediction in critically ill patients. This study aims to assess the performance of the SAPS-3 in a contemporary cohort of intensive care unit (ICU) patients in Mexico.

Methodology: Data were obtained from a prospective database, covering admissions from September 2009 to May 2024. We evaluated the discriminative ability and calibration of SAPS-3 for mortality prediction using the area under the receiver operating characteristic curve (AUC) and the Hosmer-Lemeshow goodness-of-fit test. This study adhered to the guidelines for transparent reporting of multivariable prediction models for individual prognosis or diagnosis (TRIPOD).

Results: A total of 5,033 patients were included, with a mean age of 63 ± 18 years. In-hospital mortality was 1,211 (24.06%). The SAPS-3 score had a mean of 54 ± 19, corresponding to an estimated mortality of 1,560 (31.0%). For the entire cohort, the SAPS-3 demonstrated an AUC of 0.801 (95% confidence interval (CI), 0.787-0.815), and χ^2 ^= 31.2, *P* < 0.001. In the subset of patients cared for during the COVID-19 pandemic, the AUC was 0.791 (95% CI, 0.759-0.823), and χ^2 ^= 18.4, *P* < 0.02.

Conclusions: Validation studies of SAPS-3 across diverse ICU populations consistently report good discriminative ability but limited calibration. This is the first study in Mexico with a large ICU sample, affirming that while SAPS-3 provides reliable discrimination, it tends to overestimate mortality risk.

## Introduction

Mortality prediction models are highly valued for their ability to adjust for variations in intensive care unit (ICU) patient populations and healthcare practices, making them indispensable tools in modern critical care [1]. Among their diverse applications, these models serve as benchmarking instruments, allowing ICUs to compare observed versus predicted mortality rates, which aids clinicians in identifying care gaps and potential areas for quality improvement. Additionally, the discriminative power of these models, distinguishing high-risk from low-risk patients, enables their use in resource allocation and clinical prioritization [2].

Since its introduction, the Simplified Acute Physiology Score-3 (SAPS-3) has remained one of the most widely adopted scoring systems for mortality prediction in critically ill patients worldwide [3-6]. SAPS-3 was developed as an enhancement over earlier versions, integrating a wide range of physiological and clinical parameters collected upon ICU admission.

However, despite its widespread use, predictive models such as SAPS-3 require rigorous external validation across diverse populations to ensure accuracy and reliability in varied geographic and clinical settings. Frequent recalibration is also necessary, as model performance may deteriorate over time due to advancements in medical diagnostics and treatments [7].

In Mexico, there is a marked shortage of studies validating SAPS-3 or similar prognostic models within local ICU populations, where unique healthcare conditions and patient profiles may pose specific challenges. This need is particularly relevant given that demographic, genetic, and epidemiological differences may distinguish the Mexican ICU population from those in which SAPS-3 was initially validated. Therefore, external validation in this setting is essential to determine whether SAPS-3 maintains its predictive accuracy and calibration.

This study aims to evaluate the external validity of SAPS-3 in a large cohort of ICU patients in Mexico.

## Materials and methods

Sources of data

This study is an analysis of prospectively collected data from the BASUTI database [8]. The accrual period for patient data began on September 1, 2009, and concluded on May 31, 2024, with follow-up ending at the close of the study period. Follow-up ended uniformly for all patients.

BASUTI is a computerized database established at our center in 1995. ICU medical staff, primarily critical care medicine residents, are responsible for data entry in real-time, a practice that has not changed over the past 15 years. The database captures key elements, including demographic information, pre-admission functional status, comorbidities, admission source, disease severity scores, organ failures, procedures performed, complications, admission and discharge diagnoses, as well as ICU and in-hospital outcomes.

Quality control measures for BASUTI data include: (1) weekly audits to ensure all patients are entered and data is complete through hospital discharge; (2) weekly random record reviews by senior residents with at least one year of data entry experience, overseen by ICU physicians, to verify data consistency, discrepancies found being resolved at that time and corrections undergoing a secondary review at the following weekly audit; (3) software restrictions that prevent the entry of inconsistent or implausible data such as column filters, drop-down lists, and alerts for violations of fixed ranges (as per example on age >100 years) or negative values (days of hospital stay, ICU stay, etc.); and (4) SAPS-3 calculations performed using an online calculator.

Participants 

Adult patients admitted to the 12-bed ICU of a tertiary-level reference teaching hospital in Mexico City were included in the study. This private-sector facility provides comprehensive critical care through an interdisciplinary ICU, admitting patients with a wide range of conditions, except for those with cardiac pathologies, who are managed in the center's coronary care unit.

The ICU is staffed by intensivists who provide continuous 24/7 coverage. All treatments are standardized following international guidelines, and care processes are systematically documented. The center is certified by Accreditation Canada’s Health Standards Organization (HSO), ensuring adherence to rigorous quality standards in healthcare delivery.

Outcomes and predictors

The primary outcome of this study was in-hospital mortality, defined as death before discharge. SAPS-3 scores were calculated using the standard equation, which comprises 20 variables and 61 items organized into three subgroups (or boxes). To calculate SAPS-3, clinical variables (box 3) were collected from the first hour of ICU admission, directly from the patient charts, to achieve a less biased assessment of outcomes and predictors, as ensuring a truly blinded assessment is not possible. Patient characteristics before admission (box 1) and pre-ICU circumstances (box 2) were also obtained from clinical records and entered into an online calculator on the day of admission. The resulting SAPS-3 score and estimated probability of death were then recorded in the BASUTI database.

Sample size and missing data

The study included the full cohort from the 15 years to minimize selection bias, excluding cases of readmissions and transfers to other hospitals, and those <16 years-old. SAPS-3 scores for this study were taken directly from prior database entries. A minimal number of records (37 patients, 0.67%) had missing predictors or outcome data and were therefore excluded from the analysis. For the remaining patients, no missing values were observed, as the BASUTI database designates a record as complete only when all required fields are filled. Missing data were considered as *missing completely at random*, as we found the missingness was completely unrelated to both observed and unobserved values, didn’t follow any discernible pattern, and were considered independent of any information present in our dataset.

Statistical analysis

Data are presented as means ± standard deviation or as medians with interquartile ranges for continuous variables, and as absolute and relative frequencies or percentages for categorical variables. To evaluate the calibration of SAPS-3, the Hosmer-Lemeshow goodness-of-fit test was applied using a standard configuration by grouping the observations into ten risk groups (risk deciles), grouping the 10% of observations with the lowest predicted probabilities, and so on, consecutively until grouping the 10% of observations with the highest probabilities. Discrimination was assessed using the area under the Receiver Operating Characteristic (ROC) curve (AUC). AUC confidence intervals (CI) were computed using the DeLong method. Statistical analyses were conducted using SPSS version 25 (IBM Corp., Armonk, NY), with a significance level set at *P* < 0.05. This study adhered to guidelines for transparent reporting of multivariable prediction models for individual prognosis or diagnosis (TRIPOD).

Risk groups

We evaluated SAPS-3 calibration and discrimination across the 15-year study period. Additionally, a subgroup analysis was conducted to determine whether the model’s calibration and discrimination varied during the COVID-19 pandemic care period at our center, specifically examining data from 2020 to 2022, given that these years coincide with the beginning of the epidemiological waves of the pandemic in Mexico as well as with the decline in cases that led to its end after the introduction of the vaccine. Risk deciles were recalculated within this subgroup.

Validation data vs. original data 

The validation criteria applied in this study are consistent with those used in the original SAPS-3 model. Like the original SAPS-3 cohort, this analysis included individuals aged 16 years and older admitted to the ICU, excluding incomplete records and ICU readmissions (only the first admission was considered). Age distribution was similar to that in the original cohort (median 63 years); nevertheless, our sample included a higher proportion of women (46% vs. 39%). Our cohort also comprised both medical and surgical patients, with a greater percentage admitted from the emergency room (40%) vs. the original SAPS-3 cohort, in which 38.5% were admitted from the operating room. Both cohorts had the same proportion of patients undergoing emergency surgery (17%). Our cohort exhibits slightly lower in-hospital mortality (24% vs. 28%). 

Ethical approval 

This study was approved by the local Ethics and Health Research Committees (registration number ENS-2024-T051). All procedures strictly adhered to the ethical principles outlined in the Declaration of Helsinki, ensuring full compliance with confidentiality standards and data protection regulations.

## Results

Participants

**Figure 1:**
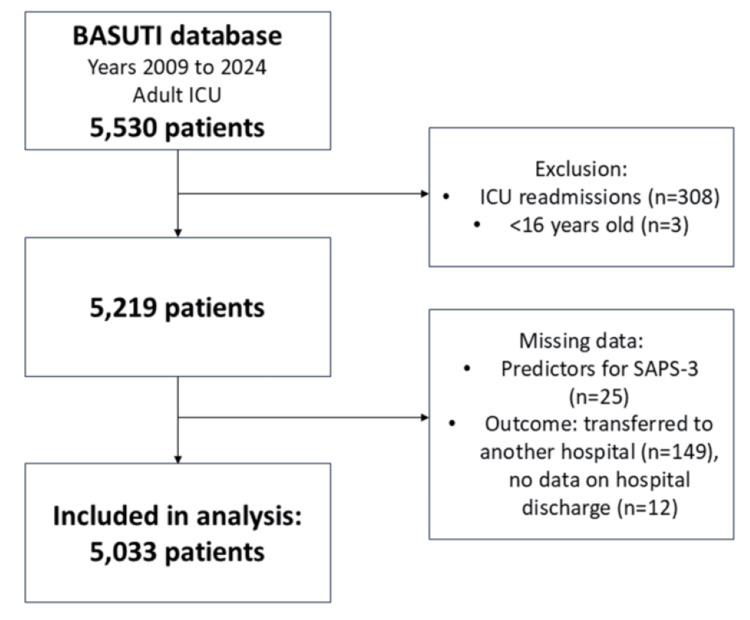
Flow diagram showing the number of excluded patients according to exclusion criteria. SAPS-3, Simplified Acute Physiology Score-3

A total of 5,530 patients were recorded in the BASUTI database during the study period. Following the application of exclusion criteria, 5033 subjects were included in the final analysis. Figure 1 presents a flow diagram illustrating patient selection.

Patient characteristics and outcomes are summarized in Table 1. The mean age of the cohort was 63 ± 18 years, with 2313 (46%) patients being female. Forty percent of patients were admitted from the emergency room, and 17% were admitted after emergency surgery. The observed in-hospital mortality was 1,211 (24.06%). The mean SAPS-3 score was 54 ± 19, corresponding to a predicted mortality of 1,560 (31.0%), calculated using the standard equation.

**Table 1:** Demographic and outcome characteristics of the included patients. SD, standard deviation; SOFA, Sequential Organ Failure Assessment; IQR, interquartile range; SAPS-3, Simplified Acute Physiology Score-3; ICU, intensive care unit

Variables	n	%
Number of patients	5,033	100
Female	2,313	46
Male	2,720	54
Age, years (mean ± SD)	63	18
SOFA (median, IQR)	5	2-8
SAPS-3 score (mean ± SD)	54	19
Location before ICU admission		
Operating room/recovery room	1,374	27.3
Emergency room	2,034	40.4
Ward	1,422	28.3
Other	203	4
Number of organ failures during ICU stay according to the SOFA score		
Cardiovascular	2,826	56.1
Respiratory	3,022	60
Renal	2,029	40.3
Liver	1,140	22.7
Hematological	1,422	28.3
Neurological	1,714	34.1
Reason for ICU admission		
Failure of one or more major organ systems	3,433	68.2
Risk of organ failure	713	14.2
Postoperative care in high-risk surgical patients	864	17.2
Missing	23	0.5
Surgical status		
Emergency surgery	869	17.3
Cardiac surgery	245	4.9
Burns	14	0.3
Invasive mechanical ventilation	2,813	55.9
Non-invasive mechanical ventilation	749	14.9
ICU mortality	810	16.1
In-hospital mortality	1,211	24.06
COVID-19 diagnosis	389	7.7

Model performance

**Figure 2:**
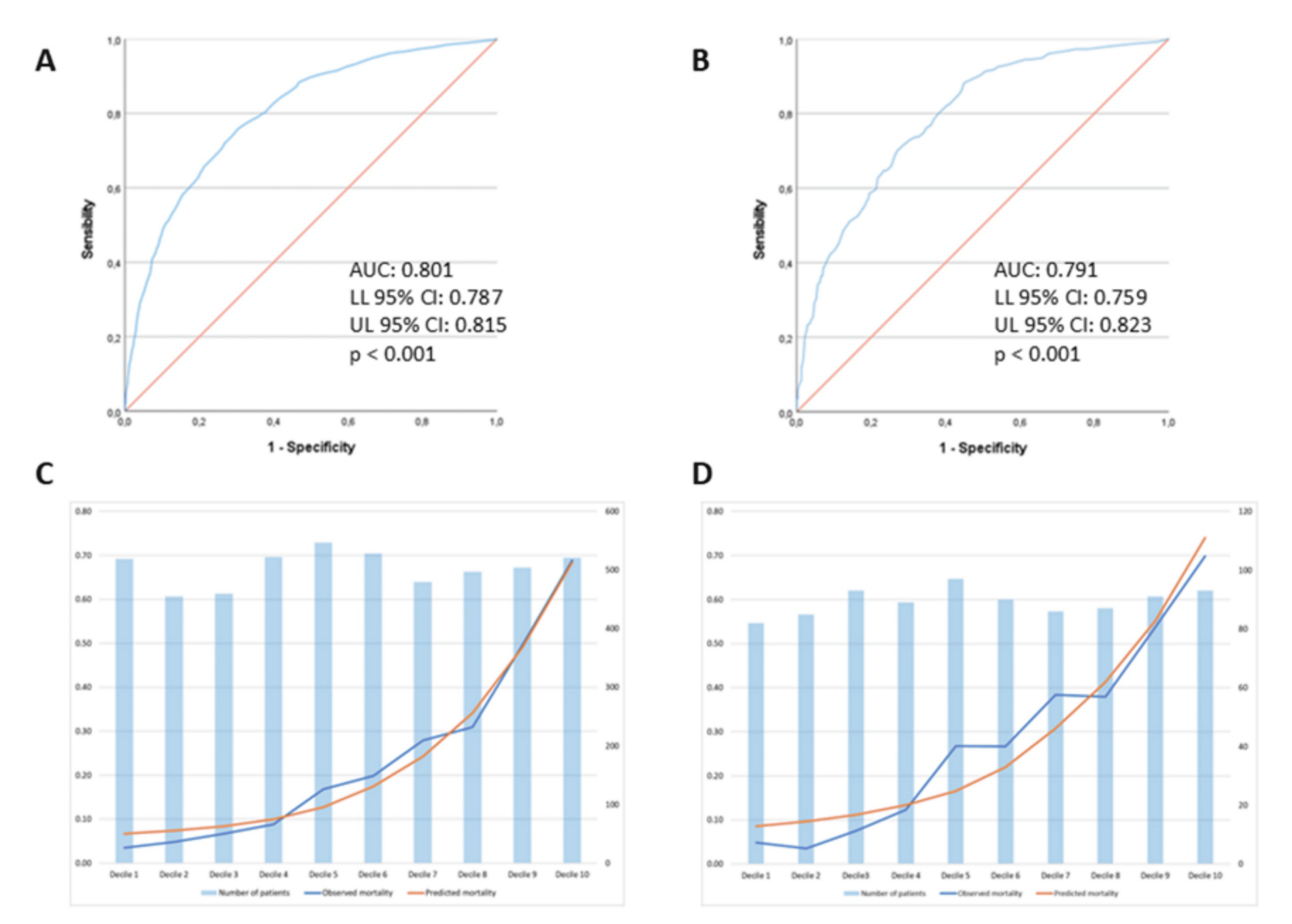
Receiver operating characteristic (ROC) curves for the entire sample and the COVID-19 care period. (A and B) ROC curves for the entire sample and the COVID-19 care period cohort, respectively. (C and D) Calibration curves for the full sample and the COVID-19 care period subgroup, respectively, showing the predicted risk of hospital mortality, the observed hospital mortality rate, and the corresponding number of patients per decile. Deciles 5, 6, and 7 exhibit higher observed mortality during the COVID-19 period, which results in the model overestimating mortality less compared with its performance in the full sample.

Model performance for the entire cohort demonstrated an AUC of 0.801 (95% CI, 0.787-0.815), and χ2 = 31.2, *P* < 0.001. In the subset of patients admitted during the COVID-19 pandemic (years 2020 to 2022), model performance yielded an AUC of 0.791 (95% CI, 0.759-0.823), and χ2 = 18.4, *P* < 0.02. Tables 2-3 display the calibration table for the entire sample and the COVID-19 care period subgroup, respectively. The ROC curves and calibration curves for the entire cohort and the COVID-19 care period subgroup are illustrated in Figure 2.

**Table 2:** Goodness of fit of the SAPS-3 standard equation for the entire sample (Hosmer–Lemeshow test: χ² = 31.2, P < 0.001). SAPS-3, Simplified Acute Physiology Score-3

Probability of death	Observed deaths	Predicted deaths	Observed survivors	Predicted survivors
Decile 1	18	34.55	501	484.45
Decile 2	22	33.52	433	421.48
Decile 3	31	38.44	429	421.56
Decile 4	46	52.34	476	469.66
Decile 5	92	69.77	455	477.23
Decile 6	105	92.16	423	435.84
Decile 7	134	116.7	346	363.3
Decile 8	154	169.83	343	327.17
Decile 9	250	247.18	254	256.82
Decile 10	359	356.51	162	164.49

**Table 3:** Goodness of fit of the SAPS-3 standard equation for the COVID-19 period cohort (Hosmer-Lemeshow χ² = 18.4, P < 0.02). SAPS-3, Simplified Acute Physiology Score-3

Probability of death	Observed deaths	Predicted deaths	Observed survivors	Predicted survivors
Decile 1	4	7.03	78	74.97
Decile 2	3	8.19	82	76.81
Decile 3	7	10.4	86	82.6
Decile 4	11	11.93	78	77.07
Decile 5	26	16.12	71	80.88
Decile 6	24	19.81	66	70.19
Decile 7	33	26.48	53	59.52
Decile 8	33	35.92	54	51.08
Decile 9	49	50.28	42	40.72
Decile 10	65	68.85	28	24.15

## Discussion

Statement of principal findings

Currently, SAPS-3 is among the most widely utilized predictive models in ICUs worldwide. Our findings indicate that the standard SAPS-3 equation demonstrates good discriminative capacity but tends to overestimate mortality. When assessing performance across various scenarios, SAPS-3, like many other predictive models, often shows poor calibration [9]. Predictive models that lack discriminative power offer limited clinical value; however, even with acceptable discrimination, a model’s utility heavily relies on its calibration, which represents its accuracy in specifying the probability of an outcome [10]. The statistically significant Hosmer-Lemeshow test found in this work denotes the model's poor calibration.

Strengths and limitations

This study has several strengths, including prospective data collection, a large sample size, and a reduced number of missing data regarding hospital outcomes. Rigorous quality controls applied to the BASUTI database contribute to a high degree of data accuracy. The case mix presented in this study likely differs from those in previous SAPS-3 validation studies, marking this as the first validation study of a mortality prediction model in a large Mexican ICU population. However, several limitations must be acknowledged. First, we will consider the temporal bias. It is unclear whether SAPS-3 would have demonstrated better calibration if applied to a cohort closer to its development, adding residual confounders that we didn´t consider. Nonetheless, this contemporary sample spans 15 years, allowing insight into the model’s performance over time. Second, as this study was conducted at a single center, the generalizability of the findings is limited; the observations and comparisons pertain only to the patient population of the authors' institution. Finally, minor differences exist between the customization and validation datasets, with our cohort exhibiting slightly lower in-hospital mortality (24% vs. 28%) despite similar ICU mortality rates.

Interpretation within the context of the wider literature

The decision to apply a specific scoring system in a given clinical scenario or population depends on the model’s performance and the completeness of data required for its calculation. The validation and predictive accuracy of mortality models can vary substantially across different contexts, and the optimal model for guiding clinical care, advancing research, and evaluating ICU performance remains undetermined [11,12].

Several validation studies of SAPS-3 have been conducted across diverse populations. A recent study in a large, contemporary Brazilian cohort supports the use of SAPS-3 for ICU benchmarking, showing that the standard equation accurately predicts patient outcomes [6]. However, studies have reported varying results regarding SAPS-3’s predictive accuracy [13]. In some populations, for example, the SAPS-3 model demonstrated slightly better calibration and overall performance compared to the MPM0-III (Mortality Probability Model at admission, version III) and APACHE-II (Acute Physiology and Chronic Health Evaluation II) models at admission [14].

A systematic review of 28 external validation studies of SAPS-3 revealed that the model's discrimination was generally very good to excellent. Nonetheless, calibration tests indicated significant deviations from perfect calibration in the majority of these studies, with statistically significant calibration issues in over half, particularly in larger studies [15]. Additionally, this review found that SAPS-3’s customized equations did not outperform the standard equation; this outcome is expected, as both the standard and customized equations produce nearly identical predictions across the full range of SAPS-3 values [4].

Differences in case mix were noted between the original SAPS-3 cohort and our validation sample. Although the age distribution of patients in the BASUTI sample was similar to that in the original cohort, our sample included a higher proportion of women (46% vs. 39%). Our cohort comprised both medical and surgical patients, with a greater percentage admitted from the emergency room or general wards, unlike the original SAPS-3 cohort, in which approximately 40% of patients were admitted directly from the operating room. A consistent feature between the cohorts was the proportion of patients undergoing emergency surgery, which remained at 17%.

The COVID-19 pandemic care period accounted for approximately one-fifth of the study period, representing an unprecedented challenge for ICUs and healthcare personnel. Overestimation of mortality observed in the overall sample was most pronounced in subgroups of patients with higher illness severity. During the COVID-19 pandemic, patients often experienced non-ideal conditions for optimal management, leading to increased mortality rates. This may partly explain the closer alignment between observed and predicted mortality in the COVID-19 care period calibration curve, thereby improving SAPS-3 calibration during this specific period.

Implications for policy, practice, and research

Our findings highlight concerns regarding the ongoing utility of SAPS-3 in our patient population, suggesting a need for recalibration to enhance its accuracy. Newer predictive models available offer similar calibration to SAPS-3 but with less data input [16,17], making them a viable alternative given the limitations of traditional models. Although not designed to compete with existing prognostic scores, rather to better describe organ dysfunction in the ICU population, the recent introduction of the Sequential Organ Failure Assessment-2 (SOFA-2) score is supported by good predictive validity [18]. Machine learning approaches also show significantly improved performance over conventional predictive models by utilizing only a few vital signs recorded over brief periods [19,20]. Automation of data analysis and collection through machine learning holds promise for critical care, though its impact on clinical decision-making requires further evaluation in prospective trials [1]; additionally, modern datasets used in these models may lack sufficient representation of demographic groups historically underrepresented in biomedical research, an issue that needs addressing to ensure broader applicability [10].

## Conclusions

Several validation studies of SAPS-3 for mortality prediction in ICU patients have been conducted across diverse populations, consistently demonstrating good discriminative ability but limited calibration. This study is the first to evaluate SAPS-3 in a large Mexican cohort, revealing that while SAPS-3 effectively discriminates outcomes, it tends to overestimate mortality. 

SAPS-3 overestimation of mortality was most pronounced in subgroups with more severe illness, as well as calibration improved during the COVID-19 care period with a closer alignment between observed and predicted mortality. These findings underscore the need for recalibration of SAPS-3 in this population, consideration of more recently developed models, and further advancements in implementing innovative tools for outcome prediction.
